# Mitochondrial Remodeling and Impaired Bioenergetics Define Neuronal Vulnerability in Fragile X-Related Disorders

**DOI:** 10.3390/antiox15101295

**Published:** 2026-10-05

**Authors:** Benedetta Niccolini, Martina Grandi, Veronica Nobile, Cecilia Pucci, Francesco Boldrin, Maria Elisabetta Clementi, Patrizia Bottoni, Alessia Riente, Pietro Chiurazzi, Giovanni Neri, Maurizio Genuardi, Alessandra Baracca, Giuseppe Maulucci, Elisabetta Tabolacci, Valentina Giorgio

**Affiliations:** 1 Section of Medical Genetics, Department of Life Sciences and Public Health, Catholic University of the Sacred Heart, 00168 Rome, Italy; niccolini.bnd@gmail.com (B.N.); veronicanobile88@gmail.com (V.N.); c.pucci91@yahoo.it (C.P.); pietro.chiurazzi@unicatt.it (P.C.); giovanni.neri43@gmail.com (G.N.); maurizio.genuardi@unicatt.it (M.G.); 2 Complex Operational Unit of Medical Genetics, 00168 Rome, Italy; 3 Fondazione Policlinico Universitario A. Gemelli IRCCS, 00168 Rome, Italy; patrizia.bottoni@unicatt.it (P.B.); alessia.riente@unicatt.it (A.R.); giuseppe.maulucci@unicatt.it (G.M.); 4 Department of Biomedical and Neuromotor Sciences, University of Bologna, 40126 Bologna, Italy; martina.grandi11@unibo.it (M.G.); alessandra.baracca@unibo.it (A.B.); valentina.giorgio4@unibo.it (V.G.); 5 Department of Biology, University of Padua, 35121 Padua, Italy; francesco.boldrin@unipd.it; 6 Istituto di Scienze e Tecnologie Chimiche “Giulio Natta” SCITEC-CNR, Largo Francesco Vito 1, 00168 Rome, Italy; elisabetta.clementi@scitec.cnr.it; 7 Department of Translational Medicine and Surgery, Catholic University of the Sacred Heart, 00168 Rome, Italy; 8 Metabolic Intelligence Lab, Department of Neuroscience, Catholic University of the Sacred Heart, Largo Francesco Vito, 1, 00168 Rome, Italy

**Keywords:** fragile X syndrome, fragile X-related conditions, *FMR1* gene, mitochondria, cyclophilin D, neurodegeneration

## Abstract

Fragile X syndrome (FXS) and related conditions associated with *FMR1* gene alterations have increasingly been linked to mitochondrial dysfunction, although most available evidence derives from peripheral cell models. Whether these abnormalities also occur in disease-relevant neuronal populations remains largely unknown. Building on our previous observations in patient-derived fibroblasts, we investigated mitochondrial structure and function in neurons differentiated from induced pluripotent stem cells (iPSCs) generated from individuals with FXS and carriers of unmethylated full mutations, using matched healthy control lines. Mitochondrial integrity, bioenergetic properties, and network organization were evaluated to determine whether *FMR1*-associated mitochondrial defects are preserved in a neuronal context. Mitochondrial abnormalities previously identified in fibroblasts were recapitulated in iPSC-derived neurons. These findings demonstrate that impaired mitochondrial dynamics and function are conserved across different cell types, indicating that mitochondrial dysfunction represents a relevant cellular feature of FXS and related *FMR1*-associated conditions. Our results indicate that mitochondrial dysfunction is a shared feature of *FMR1*-related neuronal models, while the underlying patterns of mitochondrial remodeling are influenced by the specific *FMR1*-associated condition. These results support a role for mitochondrial alterations in *FMR1*-related neuronal dysfunction and provide a basis for the further investigation of the mechanisms linking mitochondrial homeostasis to the pathophysiology of FXS and related conditions.

## 1. Introduction

Fragile X-related disorders (FXDs) comprise a heterogeneous group of inherited conditions caused by abnormal expansions of a CGG trinucleotide repeat within the 5′ untranslated region (5′UTR) of the *FMR1* gene located at Xq27.3 [1,2,3]. The size and epigenetic status of the CGG expansion critically determine *FMR1* expression and the levels and functionality of the fragile X messenger ribonucleoprotein 1 (FMRP) [4]. Among FXDs, fragile X syndrome (FXS; OMIM#300624) represents the most common monogenic cause of intellectual disability and is frequently associated with autism spectrum disorders [4]. FXS typically arises from full CGG repeat expansions exceeding 200 repeats, which undergo epigenetic silencing through DNA methylation, resulting in a profound reduction in or complete loss of FMRP expression. In contrast, carriers of premutation alleles (PM; 56–200 CGG repeats) display a distinct molecular and clinical profile and are at risk of developing fragile X-associated tremor/ataxia syndrome (FXTAS; OMIM#300623). Notably, a subset of individuals carrying full mutation alleles that escape methylation, referred to as unmethylated full mutations (UFMs), retain residual FMRP expression and often present with milder or near-normal phenotypes [5].

FMRP is a key translational regulator of neuronal mRNAs involved in synaptic function, neuronal plasticity, and genome stability, thereby playing a central role in neuronal maturation and cognitive development [6]. Accordingly, the disruption of FMRP expression or activity leads to widespread alterations in neural function. At the subcellular level, multiple independent studies have shown that FMRP deficiency compromises mitochondrial integrity, resulting in the fragmentation of the mitochondrial network and impaired morphology [7,8,9]. The mitochondrial permeability transition pore (PTP) is a key regulator of cell fate whose aberrant activation has been implicated in several neurodegenerative disorders [10,11], and its activity is finely modulated by regulatory components such as ATP synthase [12,13,14,15], the adenine nucleotide translocator (ANT) [16,17], the matrix chaperone cyclophilin D [18,19] and the ATPase inhibitor IF1 [20,21], critically influencing mitochondrial bioenergetics and susceptibility to permeability transition.

Building on this evidence, in a previous study we employed patient-derived fibroblasts as a cellular model to investigate mitochondrial function and the expression of oxidative phosphorylation complexes across distinct *FMR1* mutation states [8]. These analyses revealed pronounced mitochondrial abnormalities in fibroblasts from individuals carrying PM and UFM alleles, as well as from patients with FXS. Specifically, affected cells displayed marked alterations in mitochondrial morphology, including the accumulation of aberrant donut-shaped mitochondria, together with the upregulation of key mitochondrial proteins that are involved in mitochondrial function. These defects were further associated with increased susceptibility to the Ca^2+^-dependent opening of the mitochondrial PTP and enhanced vulnerability to mitochondrial-mediated apoptotic cell death. In a set of experiments in the same study [8], premutation and UFM fibroblasts were shown to exhibit the selective overexpression of mitochondrial proteins, including superoxide dismutase 2 (SOD2), accompanied by reduced levels of reactive oxygen species and superoxide, as well as alterations in mitochondrial membrane potential [7]. Collectively, these findings identify mitochondrial dysfunction as a consistent cellular feature associated with *FMR1* mutations.

Despite these advances, it remains unclear whether such mitochondrial abnormalities extend to disease-relevant neuronal cell types. Therefore, based on the evidence obtained in patient-derived fibroblasts, the present study aimed at investigating mitochondrial structure and function in neurons derived from induced pluripotent stem cells (iPSCs) from individuals with FXS and carriers of UFM, in comparison with matched control lines. This approach was designed to determine whether *FMR1*-associated mitochondrial alterations are preserved in the neuronal context and to confirm their potential contribution to both neurodegenerative processes and neurodevelopmental alterations.

## 2. Materials and Methods

### 2.1. Cell Lines

iPSCs and their derived neurons were the same described in Brykczynska et al. [22]. They were derived from two unrelated controls (CTRL1 and 2), two UFMs derived from two unrelated individuals (UFM1 and 2) and two FXS patients (FXS1 and 2). The protocols used for maintenance and differentiation were already published [23]. Briefly, iPSCs were derived from activated T cells isolated from PBMCs using a CytoTune-iPS Reprogramming Kit (Invitrogen, Monza, Italy). iPSCs were cultured on Matrigel (Corning, NY, USA) in mTeSR medium and differentiated into post-mitotic neurons using a dual SMAD inhibition protocol [20]. Neuronal precursor cells (NPCs) were derived by seeding iPSCs in neuronal induction medium (DMEM/F12 medium with GlutamaX (Gibco, Monza, Italy) supplemented with 20% Knockout-Serum Replacement (Gibco, Monza, Italy), 0.1 mM MEM non-essential amino acids (Gibco, Monza, Italy), 0.1 mM 2-mercaptoethanol (Gibco, Monza, Italy), Pen/Strep supplement (Gibco, Monza, Italy), 10 ng/ml hFGF (Gibco, Monza, Italy), 10 μM SB 431542 (Stemgent, Waltham, MA, USA) and 1 μM LDN 193189 (Stemgent, Waltham, MA, USA) with 10 μM ROCK inhibitor. On day 10 of NPC derivation, cells were differentiated by seeding them on Matrigel and using differentiation medium (Neurobasal Medium (Gibco, Monza, Italy), supplemented with B27 and N2 (Gibco, Monza, Italy), Pen/Strep supplement (Gibco, Monza, Italy), 10 ng/mL BDNF (R&D Systems, Milan, Italy), 10 ng/mL GDNF (R&D Systems, Milan, Italy), 10 ng/ml hNT3 (R&D Systems, Milan, Italy) for the indicated number of days (indicated as number of differentiation days plus 10 days of NPC derivation). The six donor-derived cell lines (two CTRLs, two UFMs, and two FXSs) were considered independent biological replicates, with two donor-derived cell lines representing the biological units within each experimental group. Each independent neuronal differentiation was treated as an independent culture. Repeated experiments performed using the same donor-derived cell lines were considered experimental replicates and were not treated as independent biological donors. The samples were characterized prior to the following experiments, and the corresponding data are presented in Figure S1.

### 2.2. Quantification of Mitochondrial Fraction by Confocal Microscopy and Image Processing

To quantify the relative mitochondrial content, the mitochondrial fraction was measured from confocal fluorescence images as the proportion of the mitochondrial signal relative to the total cellular area. Cells (at 30 and 60 days of differentiation) were seeded in three independent Petri dishes (1 × 10^5^ cells/dish) and processed in parallel for each experimental condition. Cells were stained with MitoTracker™ Red (Thermo Fisher Scientific, Monza, Italy) to label mitochondria and with the lipophilic membrane probe Laurdan (Thermo Fisher Scientific, Monza, Italy) to visualize cellular morphology and define cell boundaries, according to the manufacturer’s instructions. Representative confocal images of Laurdan- and MitoTracker™ Red-stained neurons are provided in Supplementary Figure S2. All samples were imaged using identical acquisition settings to allow for quantitative comparison between experimental groups.

Live-cell confocal/multiphoton imaging was performed using a Nikon A1plus laser scanning confocal microscope mounted on a Nikon Ti2 inverted microscope (Nikon, Tokyo, Japan) and equipped with GaAsP detectors. Images were acquired using a Plan Apo λ 60× oil-immersion objective (NA 1.40). Imaging was conducted under controlled environmental conditions (37 °C, 5% CO_2_) using an on-stage incubation system (OKOLAB S.r.l., Pozzuoli, Italy).

Laurdan fluorescence was acquired by multiphoton excitation at 740 nm, and emission was simultaneously collected in two spectral channels corresponding approximately to 450/50 nm and 525/50 nm. These images were used to define cellular regions of interest (ROIs), including the entire neuronal soma and neuritic extensions. MitoTracker™ Red fluorescence was acquired separately using multiphoton excitation at 900 nm and collected in the 595/45 nm emission channel. Excitation and emission parameters were kept constant across all experimental groups. Images were acquired at a spatial resolution of 2048 × 2048 pixels.

For each Petri dish, 50 fields of view were randomly selected and acquired. For each experimental sample (i.e., each independent culture/Petri dish), at least 25 images were included in the quantitative analysis.

Image analysis was performed using a custom Python-based pipeline (NumPy, SciPy, scikit-image, matplotlib, v 3.14). Raw images were subjected to Gaussian and median filtering to reduce high-frequency noise while preserving mitochondrial structures. Cellular ROIs were manually or semi-automatically defined from Laurdan fluorescence images to include the entire cell body and neuritic arborization. Mitochondrial segmentation was performed on MitoTracker™ Red images using intensity-based thresholding with identical parameters applied to all images within the same experiment. Because MitoTracker™ Red accumulation may be influenced by mitochondrial membrane potential, this analysis was not interpreted as a direct measurement of absolute mitochondrial mass. Rather, it was used to quantify the relative area occupied by the MitoTracker™ Red-positive mitochondrial signal within the cellular ROI. Therefore, the resulting parameter is referred to as MitoTracker-positive mitochondrial area fraction and was interpreted together with TEM-based ultrastructural analysis and mitochondrial protein measurements.

The segmentation threshold was defined a priori and maintained constant across all experimental groups to minimize bias related to signal intensity differences.

The MitoTracker-positive mitochondrial area fraction was calculated for each image as the ratio between the area occupied by the segmented MitoTracker™ Red-positive signal and the total cellular ROI area determined from Laurdan images:

$$\mathrm{Mitochondrial}\;\mathrm{fraction}=\frac{{\mathrm{Area}}_{\left(\mathrm{mitochondria}\right)}}{{\mathrm{Area}}_{\left(\mathrm{cell}\right)}}$$

where Area_(mitochondria)_ represents the total area occupied by the segmented mitochondrial signal, and Area_(cell)_ corresponds to the total cellular ROI area determined from Laurdan images. The resulting binary masks were used to compute total cellular area for mitochondrial fraction analysis.

To exclude potential confounding effects related to differences in cell size, total cellular area was quantified independently and did not show significant differences between experimental groups. All image acquisition and analysis parameters were defined a priori and maintained constant throughout this study.

For each image, a single MitoTracker-positive mitochondrial area fraction value was calculated. These image-level measurements were retained as individual observations for visualization and statistical analysis. Measurements obtained from the two donor-derived cell lines within each experimental group were pooled for the group-level statistical comparisons. Thus, the number of plotted observations reflects the number of images analyzed and does not represent the number of independent biological donors.

### 2.3. Electron Microscopy

Patient iPSC-derived neurons (CTR, FXS, UFM) were seeded after 30 days of differentiation at 20,000 cells/well in a 24-well plate in order to reach 50% confluence. The samples were post-fixed with 1% (*v*/*v*) osmium tetroxide in 0.1 M sodium cacodylate buffer for 1 h. After three water washes, samples were dehydrated in a graded ethanol series and embedded in an epoxy resin (Sigma-Aldrich, St. Louis, MO, USA). Ultrathin sections (60–70 nm) were obtained with an Ultrotome V (LKB) ultramicrotome, counterstained with uranyl acetate and lead citrate, and viewed with a Tecnai G2 (FEI, Hillsboro, OR, USA) transmission electron microscope operating at 100 kV. Images were captured with a Veleta (Olympus Soft Imaging System, Tokyo, Japan) digital camera. Mitochondria were analyzed by counting the number of total mitochondria per image, cristae per mitochondrion, swollen mitochondria, and mitochondrial length as in [24].

### 2.4. Western Blotting Analysis

Cells (10 × 10^6^) were kept on ice for 20 min in 0.15 mL of a buffer containing 150 mM NaCl, 20 mM Tris, and 5 mM EDTA-Tris, pH 7.4, with the addition of 1% (*v*/*v*) Triton X-100, 10% (*v*/*v*) glycerol, phosphatase, and protease inhibitors. Sample buffer (Nu-PAGE™ LDS sample buffer, Invitrogen supplemented with 12.5% *v*/*v* β-mercaptoethanol) was added to supernatants, and samples were separated by polyacrylamide gel (NuPAGE™, 12% Bis-Tris, Invitrogen, Waltham, MA, USA) electrophoresis and transferred to nitrocellulose membranes. Blocking was performed with a PBS solution containing 5% (*w*/*v*) non-fat dry milk (AppliChem, Darmstadt, Germany). Antibodies for OXPHOS (OXPHOS Human WB Antibody Cocktail, RRID: AB_2756818), adenine nucleotide translocator isoform 3 (ANT, RRID: AB_2619664), citrate synthase (CS, RRID: AB_10678258), cyclophilin D (CyPD, RRID: AB_10864110), ATPase inhibitor (IF1, RRID: AB_10861497), and the β (RRID: AB_301438), b (RRID: AB_10901555), c (RRID: AB_2935765), and OSCP (RRID: AB_10887942) subunits were from Abcam (Cambridge, UK), and the one against GAPDH (RRID: AB_561053) was from Cell Signaling (Danvers, MA, USA). The antibody for SOD2 was from Enzo Life Sciences (New York, USA) (RRID: ADI-SOD-111-D). The band pixels of each replicate are normalized on the band pixels of their proper loading control. Mean pixel ratios ± SEM are shown. Western blotting band intensities were analyzed using the ChemiDoc MP system equipped with ImageLab software (version 6.1, Bio-Rad, Hercules, CA, USA) or ImageJ software (version 1.52p, RRID: SCR_003070).

### 2.5. Reactive Oxygen Species (ROS) Measurement

Cells were seeded into 96-well plates at a density of 15,000 cells per well and allowed to adhere for 24 h. Intracellular ROS levels were then measured using a commercially available fluorometric assay (DCFDA Cellular ROS Detection Assay Kit; Abcam, Milan, Italy; ab113851), according to the manufacturer’s instructions. Briefly, the non-fluorescent probe DCFDA diffuses into cells, where it is oxidized by reactive oxygen species to produce a fluorescent signal. Fluorescence intensity was recorded using a Victor™ 4 microplate reader (PerkinElmer, Waltham, MA, USA) at excitation and emission wavelengths of 485 nm and 535 nm, respectively. ROS level production was expressed as relative fluorescence units (RFU). Although DCFDA is an effective tool for general redox screening, it tracks global intracellular oxidative shifts and lacks specificity for individual reactive oxygen species.

### 2.6. Oxygen Consumption Rate

The oxygen consumption rate (OCR) in iPSC-derived neurons from FXS and UFM patients and control neurons was measured with the Seahorse XF HS Mini Analyzer using the Seahorse XFp Cell Mito Stress Test Kit (Agilent Technologies, Santa Clara, CA, USA). Briefly, after 30 days of differentiation, iPSC-derived neurons were seeded in XF HS cell culture microplates at 15,000 cells/well and maintained at 37 °C in a 5% CO_2_ humidified incubator for 24 h before OCR measurements. The number of cells per well was counted at the end of OCR measurements and remained unchanged. The day after, the growth medium was replaced with the Seahorse medium assay (supplemented with 10 mM glucose, 1 mM pyruvate and 2 mM L-glutamine, according to the manufacturer’s protocol), and cells were incubated in a CO_2_-free incubator for one hour prior to measurements to allow for temperature and pH equilibration. After an OCR baseline measurement, 1 μM oligomycin, 0.2–1 μM carbonyl cyanide-p-trifluoromethoxyphenylhydrazone (FCCP), 1 μM rotenone, and 1 μM antimycin A were sequentially added to each well. Before each experiment, a titration curve with FCCP was performed to assess the optimal FCCP concentration that maximally stimulates respiration. The acidification rate (ECAR) was simultaneously monitored with the OCR under different respiration conditions. All OCR data and ECAR data were normalized to the total cellular number determined after each experimental run.

### 2.7. Quantification of Intracellular Nrf2 Levels

Neuronal cells were seeded in 96-well plates at a density of 1.5 × 10^4^ cells per well, according to the experimental design described above. For each experimental sample, intracellular Nrf2 protein levels were quantified using a cell-based colorimetric ELISA (LifeSpan Biosciences, LSBio; Seattle, WA, USA), following the manufacturer’s instructions. Briefly, cells were incubated with primary antibodies directed against Nrf2 (rabbit anti-Nrf2) or glyceraldehyde-3-phosphate dehydrogenase (mouse anti-GAPDH), the latter used as an internal normalization control. After overnight incubation at 4 °C, horseradish peroxidase-conjugated secondary antibodies (anti-rabbit IgG for Nrf2 and anti-mouse IgG for GAPDH), supplied with the kit, were applied. The colorimetric signal was detected using a BioTek™ ELx800 microplate reader (BioTek Instruments, Winooski, VT, USA) by measuring absorbance at 450 nm. Nrf2 levels were expressed as absolute Nrf2/GAPDH ratios.

### 2.8. Statistical Analysis

Statistical analyses were performed separately for each cell type, considering the two independent donor-derived cell lines within each group. Technical replicates were treated as such and were not considered independent biological replicates. A *p* value < 0.05 was considered statistically significant.

## 3. Results

### 3.1. Atypical Mitochondrial Morphology and Differentiation-Stage-Dependent Alterations in FXS and UFM iPSC-Derived Neurons

**Figure 1:**
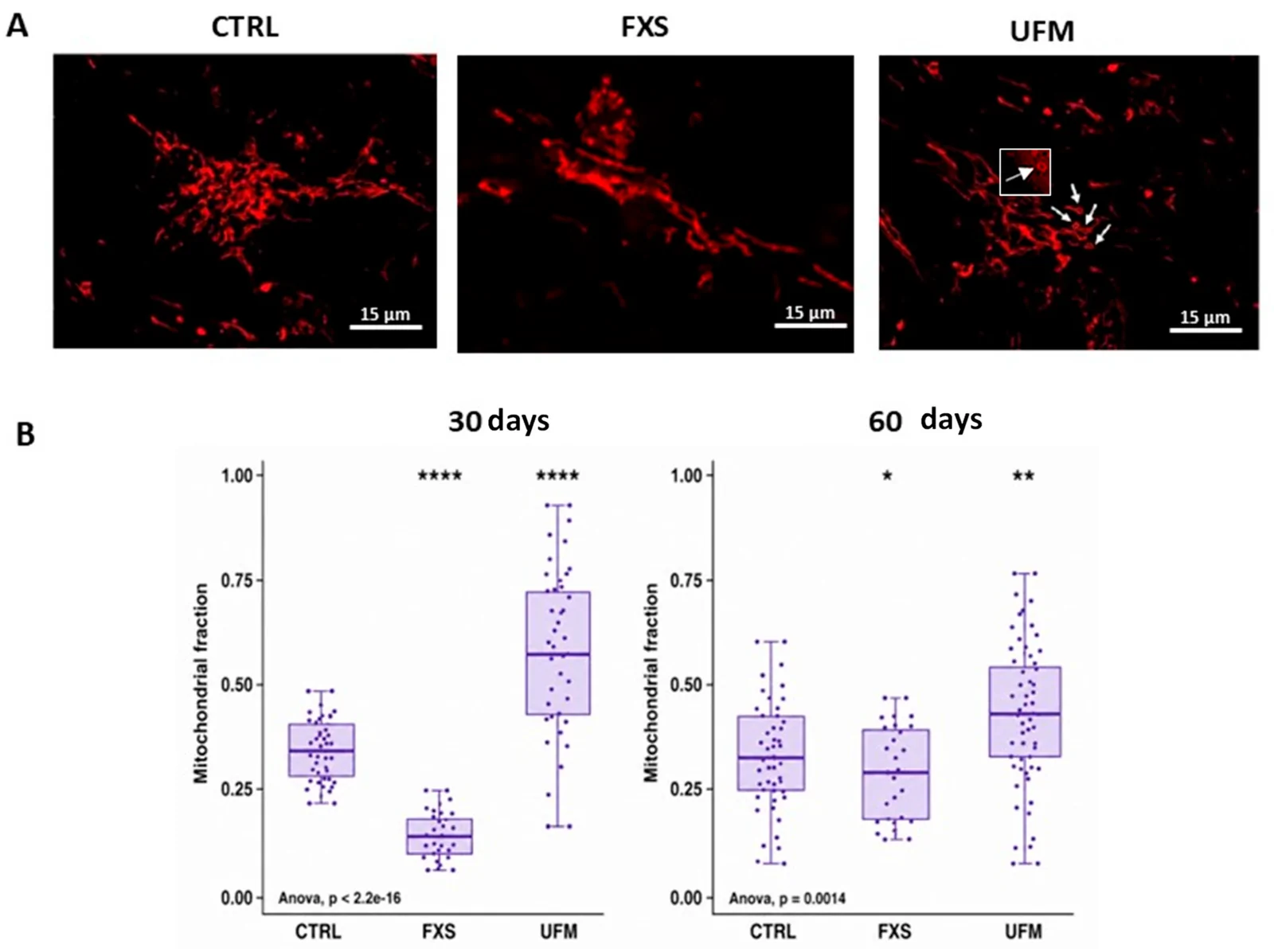
Mitochondrial morphology and the MitoTracker-positive area fraction in iPSC-derived neurons through confocal microscopy analysis. (**A**) Representative confocal microscopy images of MitoTracker™ Red-labeled mitochondria in iPSC-derived neurons from control (CTRL), FXS patients, and UFM carriers. The images are single-plane confocal images and not shown as z-stacks. Note aberrant donut-shaped mitochondria in UFM-derived neurons (white arrows and magnification). Scale bar: 15 µm. (**B**) A quantitative analysis of the mitochondrial fraction in iPSC-derived neurons at early (30 days) and later (60 days) stages of neuronal differentiation. The mitochondrial fraction was calculated as the proportion of the cellular area occupied by the MitoTracker™ Red-positive signal relative to the total cellular area defined by Laurdan staining, providing an estimate of the MitoTracker-positive mitochondrial area fraction. At least 25 images were acquired for each experimental sample. Each dot represents the mitochondrial fraction measured in an individual cell, and the plotted values were derived from multiple images acquired for each experimental sample. For statistical analysis, cell-level measurements from the two donor-derived cell lines within each group were pooled. Box plots indicate the median and interquartile range. Statistical significance is indicated in the panels (* *p* = 0.0103, ** *p* = 0.0036, **** *p* ≤ 0.0001; ANOVA post hoc Holm–Šídák).

Mitochondrial morphology in iPSC-derived neurons from FXS patients, UFM carriers, and control lines was initially investigated by confocal microscopy (Figure 1; see Figure S1 for characterization of iPSC-derived neurons). A qualitative analysis of the confocal images of adherent neurons (30 days of differentiation) stained with MitoTracker™ Red revealed mitochondrial structural abnormalities that were most evident in UFM cells and also present, although less prominently, in FXS cells, including the frequent occurrence of aberrant donut-shaped mitochondria (Figure 1A, white arrows and magnification). Notably, these morphological alterations closely recapitulate the mitochondrial defects previously described in fibroblasts derived from PM and UFM patients, supporting the presence of partially conserved mitochondrial phenotypes across different patient-derived cell types [7].

To further characterize these alterations, confocal microscopy images were quantitatively analyzed as described in Section 2. Briefly, relative mitochondrial content was estimated by measuring the area occupied by the MitoTracker™ Red-positive mitochondrial signal relative to the total cellular area defined by Laurdan membrane staining (see Figure S2 for representative images of Laurdan and MitoTracker staining in derived neurons). This approach provided an estimate of the relative cellular area occupied by MitoTracker™ Red-positive mitochondrial structures, rather than a direct measurement of absolute mitochondrial mass or organelle number. Accordingly, the confocal analysis was interpreted in combination with TEM-based quantification and biochemical assessment of mitochondrial proteins.

Quantitative analysis revealed opposite changes in the mitochondrial fraction at early stages (30 days) of neuronal differentiation in FXS and UFM neurons compared with controls (Figure 1B, left panel). Specifically, neurons derived from FXS patients exhibited a significant reduction in mitochondrial fraction, whereas neurons derived from UFM carriers showed a marked increase at the same differentiation stage. Importantly, these differences progressively diminished during neuronal maturation, resulting in a partial convergence of mitochondrial fraction values across genotypes at later stages of differentiation (60 days) (Figure 1B, right panel).

**Figure 2:**
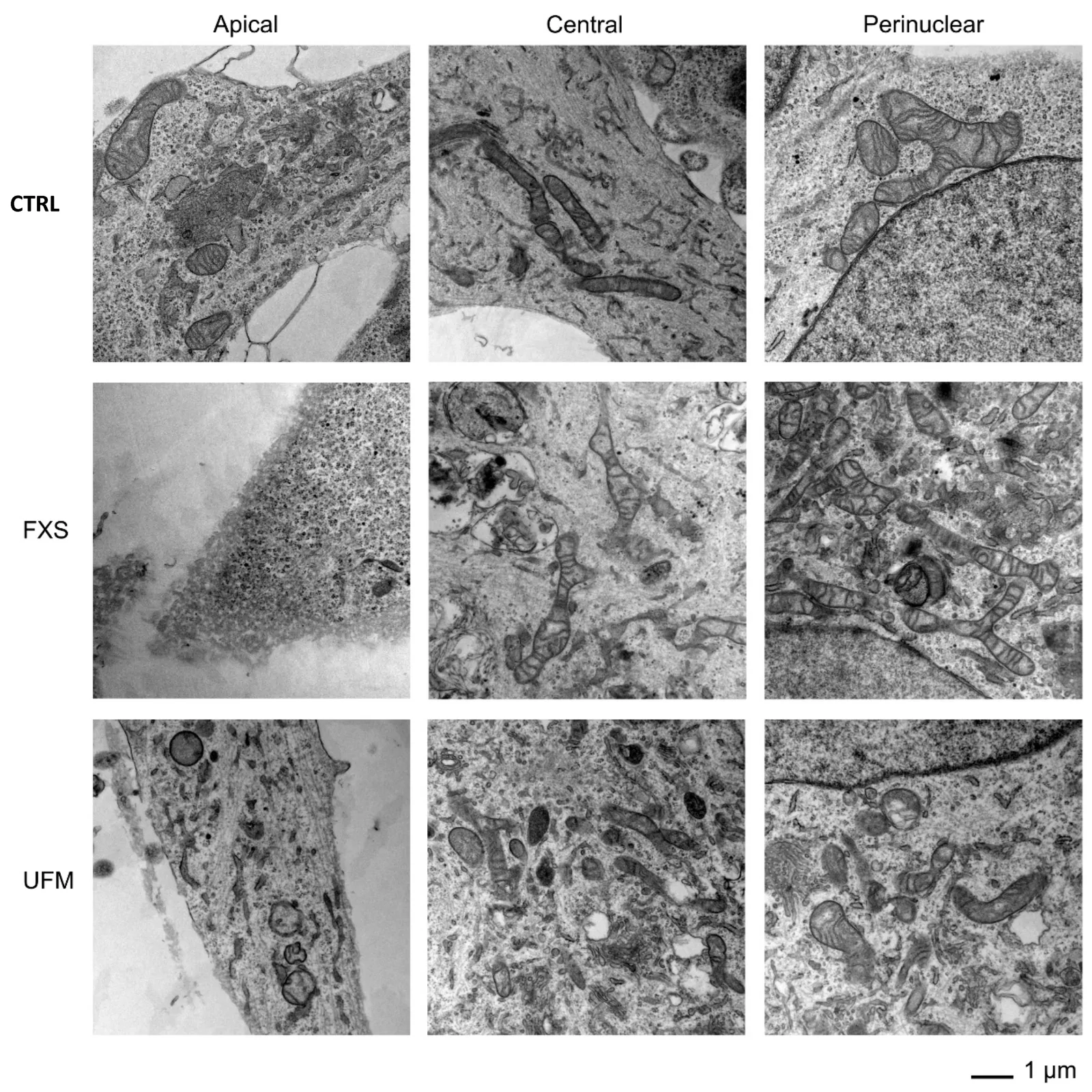
Representative transmission electron microscopy (TEM) images of iPSC-derived neurons. Representative images of fixed cells show different mitochondrial distributions in distinct neuronal compartments: apical, central, and perinuclear regions from control (CTRL), FXS patients, and UFM iPSC-derived neurons after 30 days of differentiation. Scale bars: 1 µm.

The mitochondrial morphology was further investigated by transmission electron microscopy (TEM) analysis (Figure 2). TEM imaging confirmed the presence of pronounced ultrastructural abnormalities in mitochondria from neurons derived from both FXS patients and UFM carriers (30 days), including altered shape, abnormal elongation, and the disorganization of cristae across distinct neuronal compartments, namely the apical, central, and perinuclear regions.

**Figure 3:**
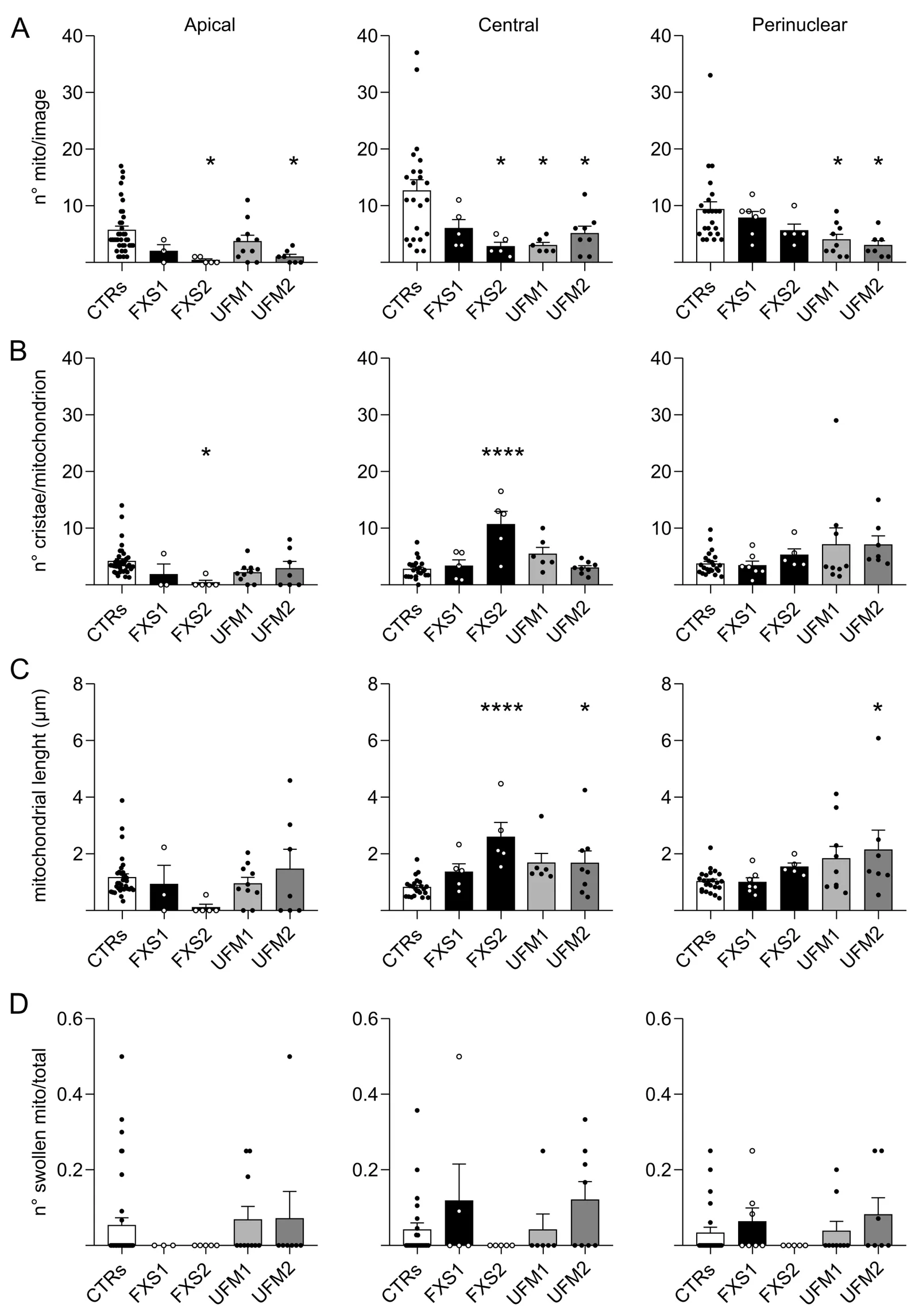
Analysis of the mitochondrial morphology of iPSC-derived neurons. The quantification of the number of mitochondria per image (**A**), the number of cristae per mitochondrion (**B**), the mean mitochondrial length (µm) (**C**) and the ratio of the number of swollen/total mitochondria (**D**) are shown. The two control cell lines were matched to the patient-derived cell lines for the respective comparisons. Data represents technical/experimental replicates obtained from two independent donor-derived cell lines per group and are presented as the mean ± SEM. Statistical significance is reported in the panels (* *p* ≤ 0.05, **** *p* ≤ 0.0001, one-way ANOVA, Dunnett’s multiple comparison test, patient vs. controls).

In addition to these structural defects, TEM analysis revealed a marked reduction in mitochondria in patient-derived neurons. The number of both FXS and UFM mitochondria per image appeared to be decreased in the apical and central regions of neuron bodies, while only UFM mitochondria were significantly decreased in the perinuclear region, compared to those of healthy donors (Figure 3A). Notably, neurons derived from UFM carriers frequently exhibited a striking depletion of mitochondria in the apical and perinuclear regions, corresponding to the compartments involved in the reception of neuronal inputs and mitochondrion–nucleus crosstalk, respectively (Figure 3). In several cases, these regions appeared largely devoid of mitochondria, highlighting a compartment-specific vulnerability in UFM neurons.

Other mitochondrial parameters were analyzed in a compartment-specific manner (Figure 3). An assessment of mitochondrial ultrastructure further revealed region-specific alterations in crista organization. A reduction in crista density, quantified as the number of cristae per mitochondrion (n° cristae/mitochondrion), was observed in the apical compartment of FXS- and UFM-derived neurons compared to controls (Figure 3B). In the central compartment, on the contrary, a higher number of cristae per mitochondrion was detected in both the FXS and UFM phenotypes relative to controls (Figure 3B). In the perinuclear compartment, crista density did not differ significantly from control neurons across any of the genotypes analyzed (Figure 3B).

Central and perinuclear compartments showed longer mitochondria in FXS and UFM phenotypes, while an opposite, although not significant, trend was revealed in the apical regions of the same phenotypes compared to controls (Figure 3C). Surprisingly, FXS and UFM phenotypes did not show higher number of swollen mitochondria in any of the neuron cellular regions, as compared to normal neurons (Figure 3D). This result might apparently seem in contrast with higher PTP sensitivity to calcium and PTP-dependent apoptosis that were shown in fibroblasts derived from FXS and UFM patients [8]. However, neurons that are subjected to higher reactive oxygen species than fibroblasts, due to their high mitochondrial function, might more rapidly undergo apoptosis upon mitochondrial swelling, and this in turn might cause an underestimation of swollen mitochondria, which are not detected in living neurons.

Overall, these findings reveal spatially restricted and genotype-specific mitochondrial defects in iPSC-derived neurons, with compartment-dependent alterations in mitochondrial abundance and crista organization.

### 3.2. Mitochondrial OXPHOS and Differentiation-Stage-Dependent Alterations in FXS and UFM iPSC-Derived Neurons

**Figure 4:**
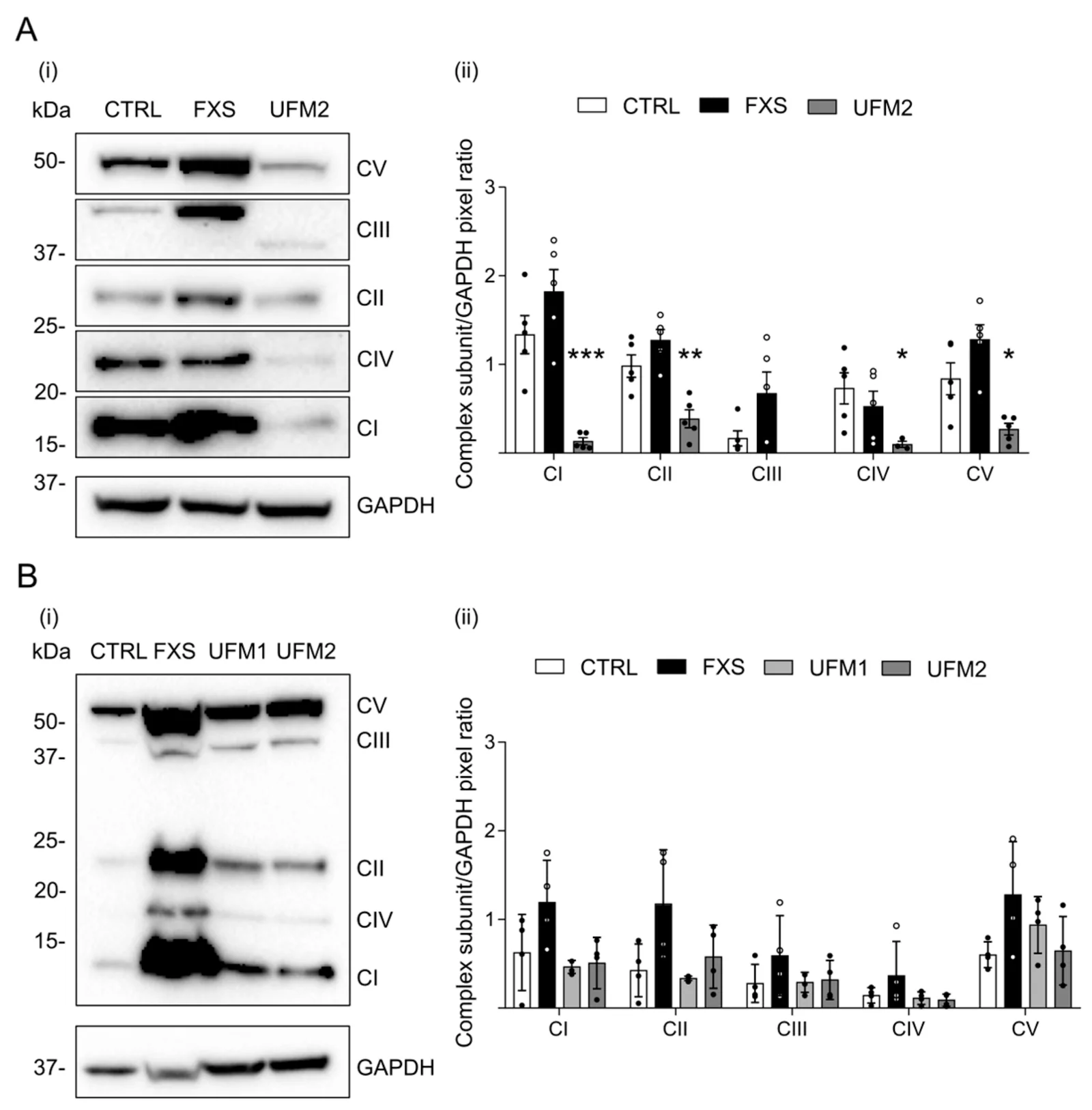
An analysis of mitochondrial OXPHOS complexes in iPSC-derived neurons. Western blotting shows OXPHOS complex subunits in CTRL, FXS and UFM patient-derived neurons at 30 (**A**) and 60 (**B**) days of differentiation. (**i**) Representative images of Western blot. The molecular marker is indicated on the left. GAPDH, glyceraldehyde-3-phosphate dehydrogenase. Histograms (**ii**) show the mean of each complex subunit/GAPDH pixel ratio. Data are expressed as the mean± SEM of at least three independent experiments performed using two donor-derived cell lines per group. (* *p* ≤ 0.05, ** *p* = 0.0061, *** *p* = 0.0006, Student’s *t* test.)

Since FXS and UFM iPSC-derived neurons showed an overall decrease in the mitochondrion number as analyzed by TEM (Figure 3A), neuron lysates from the same genotypes were analyzed through Western blotting in order to assess their OXPHOS complex composition in mitochondria at 30 or 60 days of differentiation. In FXS iPSC-derived neurons, a mild increase in OXPHOS complexes was revealed compared to controls upon 30 days of differentiation (Figure 4A), which is in line with the increased number of cristae of this genotype as revealed in the apical and central regions (Figure 3B). The higher levels in the OXPHOS complex subunits of FXS neurons were maintained up to 60 days of differentiation (Figure 4B). On the contrary, the analysis of UFM lysates showed a dramatic decrease in the OXPHOS subunit levels of iPSC-derived neurons that were differentiated for 30 days (Figure 4A), in line with the decreased number of mitochondria and a comparable number of cristae per mitochondrion than control neurons (Figure 3A,B). Importantly, the decrease in the OXPHOS content of UFM neurons was totally rescued during neuronal differentiation, thus reaching the control levels at 60 days (Figure 4B).

### 3.3. Other Key Mitochondrial Proteins and Differentiation-Stage-Dependent Alterations in FXS and UFM iPSC-Derived Neurons

**Figure 5:**
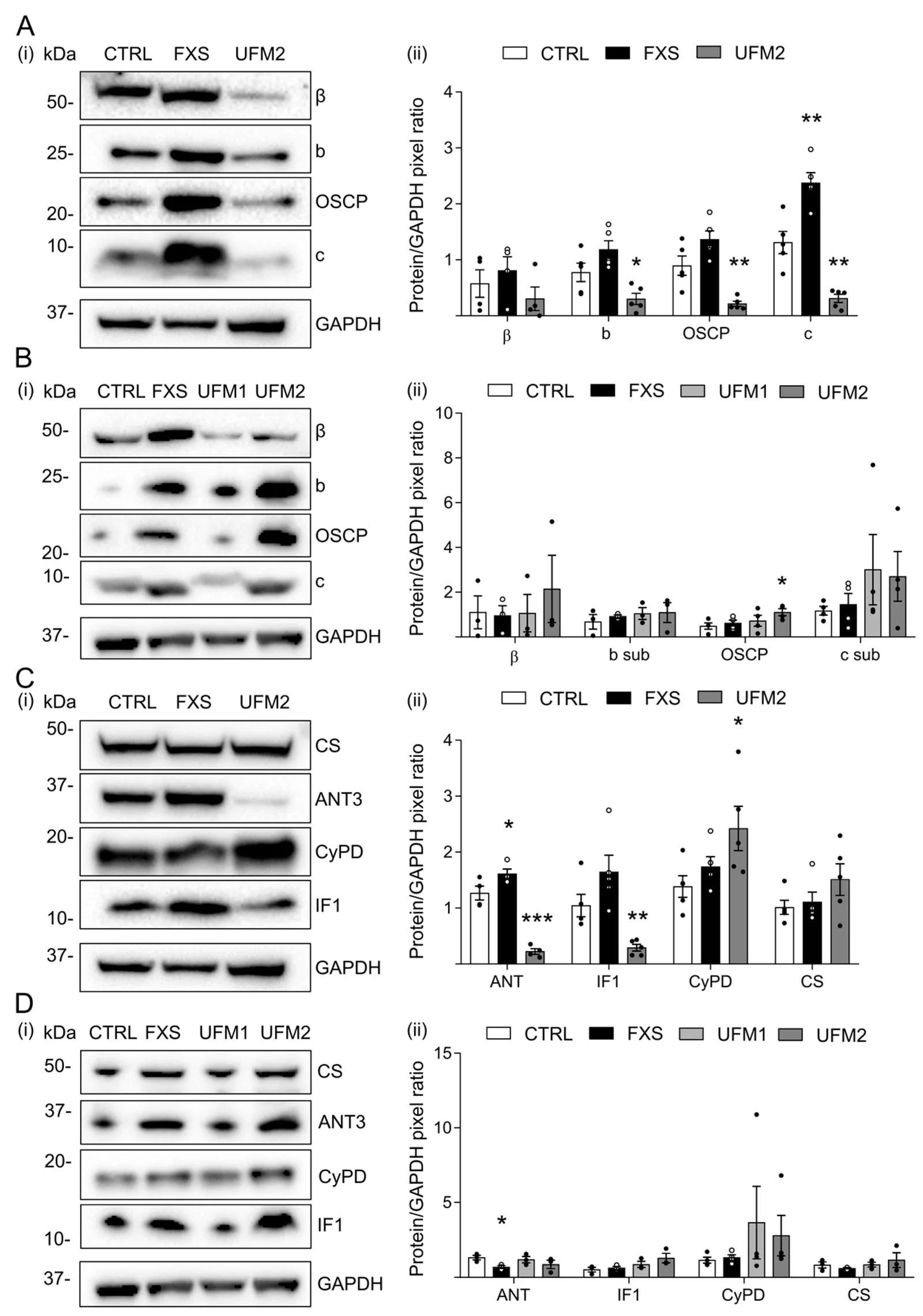
An analysis of ATP synthase subunit composition and other key mitochondrial proteins in iPSC-derived neurons. Western blotting shows the β, b, OSCP, and c subunits of ATP synthase (**A**,**B**) and CS, ANT3, CyPD, and IF1 (**C**,**D**) in CTRL, FXS and UFM genotypes at 30 (**A**,**C**) and 60 (**B**,**D**) days of differentiation (**i**). The molecular marker is indicated on the left. GAPDH, glyceraldehyde-3-phosphate dehydrogenase; CS, citrate synthase. Loading controls are repeated in panels (**A**,**C**) or (**B**,**D**) because the detected proteins derive from the same experimental run and Western blotting. Histograms (**ii**) show the mean of the protein/GAPDH pixel ratio. Data are the mean ± SEM of at least three independent experiments performed using two donor-derived cell lines per group. (* *p* ≤ 0.05, ** *p* ≤ 0.01, *** *p* = 0.0002, Student’s *t* test.)

Different ATP synthase subunits were analyzed at the same time points of neuron differentiations as described above. In FXS iPSC-derived neurons, an increase in ATP synthase subunits β, b, OSCP and c was shown upon 30 days (Figure 5A), but these levels were normalized and became comparable to those of normal (CTRL) neurons at 60 days of differentiation (Figure 5B). On the other hand, UFM iPSC-derived neurons showed decreased levels of the ATP synthase subunits at 30 days (Figure 5A) but an increase in the OSCP and c subunits at 60 days of differentiation (Figure 5B). Other key mitochondrial proteins, such as the adenine nucleotide transporter (ANT), the IF1 inhibitor of ATP synthase, and cyclophilin D (CyPD), that are involved in the modulation of the PTP [16,18,24] are differently expressed in FXS or UFM neurons at the two stages of cell differentiation. On the one hand, in FXS genotypes, ANT and CyPD were mildly upregulated at 30 days, while they decreased at 60 days of neuron differentiation (Figure 5C,D). On the other hand, in UFM cells, ANT and the inhibitor protein IF1 levels were very low, while the levels of CyPD were significantly higher than in normal neurons, at 30 days of differentiation (Figure 5C). The CyPD expression level also stayed high at 60 days of differentiation in UFM neurons (Figure 5D), suggesting higher sensitivity to the PTP opening of UFM genotypes compared to the others, at the late stage of cell differentiation.

### 3.4. FXS and UFM Neurons Exhibit Defective Mitochondrial Respiration

**Figure 6:**
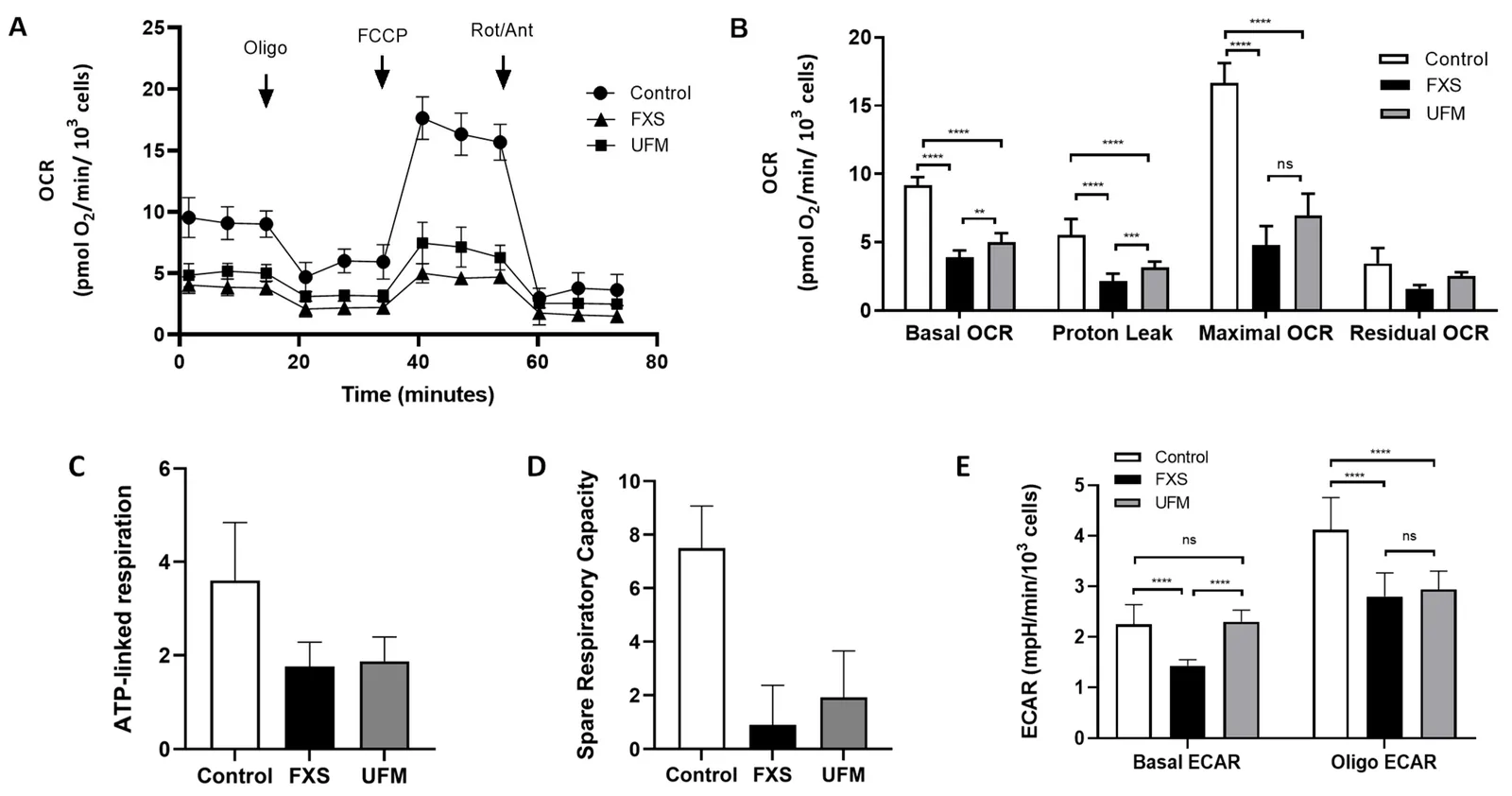
Mitochondrial respiration and glycolytic activity in iPSC-derived neurons from FXS patients and UFM carriers. Mitochondrial respiration and glycolytic flux were assessed in iPSC-derived neurons from control, UFM, and FXS neurons at 30 days of differentiation. (**A**) A time-course analysis of the oxygen consumption rate (OCR) showing basal respiration and responses to the sequential addition of oligomycin (Oligo), the mitochondrial uncoupler FCCP, and electron transport chain inhibitors rotenone and antimycin A (Rot/Ant). Data are the mean ± SEM of three independent experiments. (**B**) The quantification of mitochondrial respiratory parameters derived from the OCR profiles, including the basal OCR, proton leak-associated respiration, maximal OCR, and residual OCR following electron transport chain inhibition. Data are the mean ± SEM of three independent experiments performed using two donor-derived cell lines per group. (**** *p* < 0.0001; *** *p* < 0.001; ** *p* < 0.01; ns = not significant with *p* = 0.0862; one-way ANOVA with Tukey’s post hoc test.) (**C**,**D**) The quantification of ATP-linked respiration and spare respiratory capacity in the three different iPSC-derived neurons (control, FXS and UFM). (**E**) The extracellular acidification rate (ECAR) in the basal condition and upon the addition of oligomycin (Oligo). Data are presented as the mean ± SEM of three independent experiments performed using two donor-derived cell lines per group. (**** *p* < 0.0001, one-way ANOVA with Tukey’s multiple comparison test.)

Mitochondrial respiration was assessed in iPSC-derived neurons from FXS and UFM patients and compared with control neurons by examining metabolic activity in adherent neurons at 30 days of differentiation and using specific inhibitors or an uncoupler in order to dissect the respiratory complex contribution among the different genotypes (Figure 6). A time-course analysis of the oxygen consumption rate (OCR), shown in Figure 6A, revealed distinct respiratory profiles among the three groups. Under basal conditions, FXS- and UFM-derived neurons exhibited lower OCR values compared with control neurons. Following the addition of oligomycin, the OCR decreased in all conditions, indicating low but well-coupled oxidative phosphorylation in FXS and UFM neurons. Subsequent stimulation with the mitochondrial uncoupler FCCP induced a huge increase in the OCR in control neurons, whereas a markedly attenuated response was observed in both FXS- and UFM-derived neurons, in agreement with either a high level, although not functional, or a low level of expression in OXPHOS complexes that were revealed in FXS or UFM neurons at 30 days of differentiation (Figure 4A), respectively. Finally, the inhibition of the electron transport chain with rotenone and antimycin A reduced the OCR to similarly low levels across all groups. Consistent with the OCR time-course shown in Figure 6A, a quantitative analysis of respiratory parameters (Figure 6B) confirmed a significant reduction in the basal and maximal OCR in FXS- and UFM-derived neurons compared with controls. ATP-linked respiration and spare respiratory capacity showed a trend toward a reduction in FXS and UFM neurons compared with controls, indicating the lower capacity of oxidative phosphorylation to support ATP synthesis in FXS and UFM and a limited capacity to respond to increased energetic demands or cellular stress, respectively.

To additionally assess whether alterations in mitochondrial respiration were accompanied by changes in glycolytic activity, the extracellular acidification rate (ECAR) was analyzed over time (Figure 6E). In basal conditions, the ECAR was significantly lower in FXS but not in UFM neurons compared to controls. Although upon the oligomycin treatment all the genotypes showed an increase in glycolytic flux (ECAR) as expected, FXS- and UFM-derived neurons displayed consistently and statistically significantly (with *p* < 0.0001 for both comparisons) lower ECAR values compared with control neurons, indicating lower glycolytic capacity and possible differences in the glycolytic enzyme levels or their modulation in FXS and UFM genotypes.

Importantly, these findings differ from the previously reported data obtained in patient-derived fibroblasts [8], in which basal and maximal respiration were largely preserved in FXS and UFM cells despite the accumulation of OXPHOS complex subunits. In contrast, in the neuronal model used in this study, functional alterations in mitochondrial respiration were readily detectable, particularly under conditions of maximal respiratory stimulation. This discrepancy highlights the cell type-specific vulnerability of neuronal mitochondria and underscores the relevance of a disease-relevant neuronal model.

### 3.5. Mitochondrial Superoxide Dismutase Expression, Intracellular Reactive Oxygen Species, and Nrf2 Protein Levels in FXS and UFM iPSC-Derived Neurons

**Figure 7:**
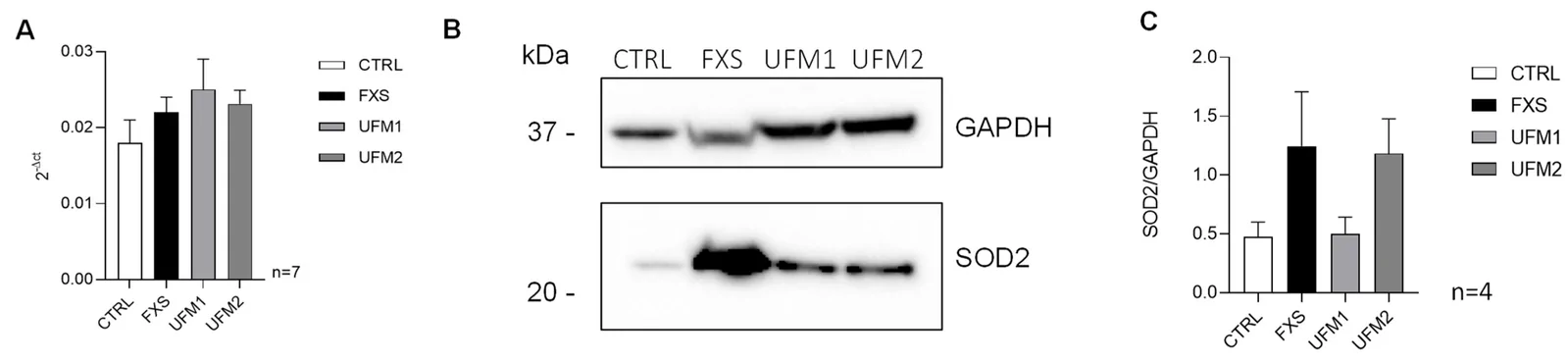
Analysis of SOD2 expression in iPSC-derived neurons. (**A**) Quantitative PCR (qPCR) analysis of SOD2 transcript levels in iPSC-derived neurons from control (CTRL), UFM carriers, and FXS patients. Data are expressed as 2^−ΔCt^ values and normalized to *GAPDH* as housekeeping gene. Data are mean ± SEM of 7 independent experiment replicates performed using two donor-derived cell lines per group. (**B**) Representative Western blotting showing SOD2 levels in iPSC-derived neurons from CTRL, FXS, UFM1 and UFM2 genotypes, with GAPDH used as loading control. Western blotting was obtained by reprobing, after stripping, membrane used for 60-day blot shown in Figure 4B. (**C**) Histogram shows quantification of SOD2 protein band normalized on GAPDH (pixel ratio). Data are presented as mean ± SEM of 4 independent experiment replicates using same donor-derived cell lines.

The transcript and protein levels of superoxide dismutase 2 (SOD2) were evaluated in iPSC-derived neurons from FXS patients, UFM carriers, and control lines (at 60 days of differentiation) by quantitative PCR (qPCR) and Western blotting analysis (Figure 7). Increased *SOD2* transcript levels in neurons derived from both UFM carriers and FXS patients compared to control neurons were revealed by qPCR analysis, although these differences did not reach statistical significance (Figure 7A). Consistent with the transcriptional trend, Western blotting analysis showed increased SOD2 protein levels in neurons derived from UFM carriers (particularly UFM2) and, more prominently, from FXS patients compared with controls. The quantification of SOD2 protein levels normalized to GAPDH confirmed an upward trend in UFM-derived neurons that did not reach statistical significance (Figure 7B). These findings partially align with previous observations in patient-derived fibroblasts [7] while revealing a distinct, cell-type-specific pattern of SOD2 regulation.

**Figure 8:**
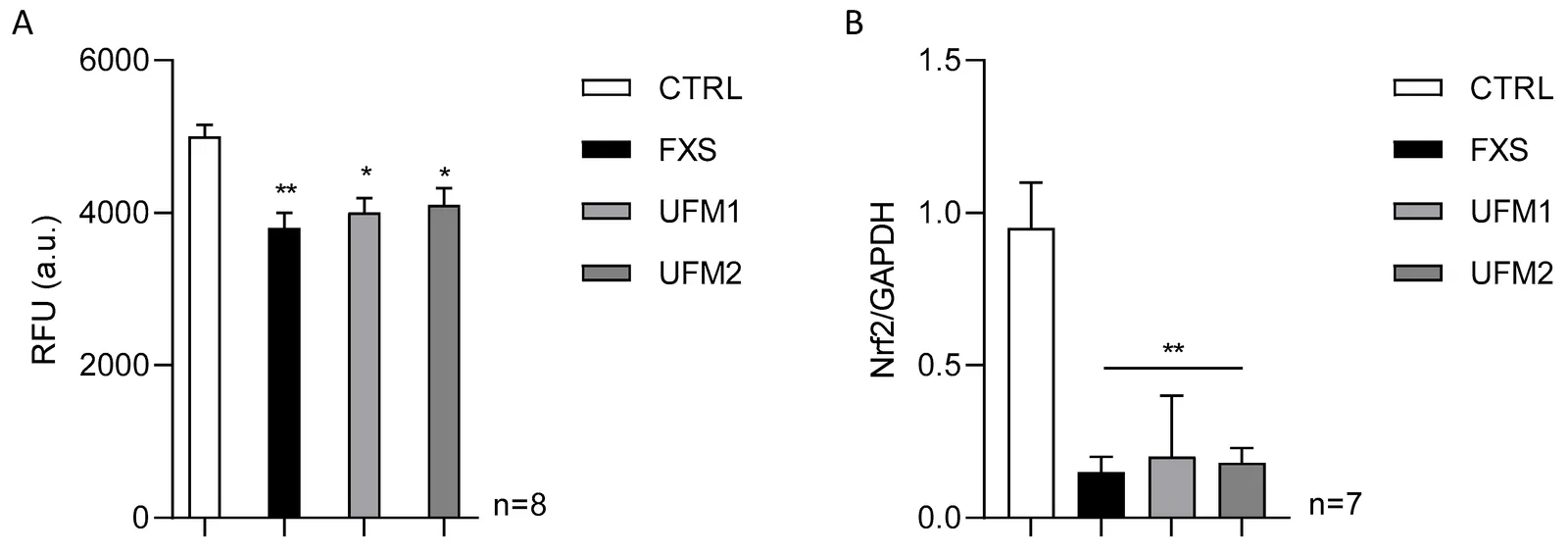
Intracellular reactive oxygen species (ROS) and Nrf2 protein levels in iPSC-derived neurons. (**A**) Intracellular ROS levels were quantified using a fluorometric assay in iPSC-derived neurons from control (CTRL), FXS patients and UFM carriers. Neurons derived from both FXS patients and UFM carriers display significantly reduced ROS levels compared with control neurons. Data are presented as the mean ± SEM of 8 independent experiment replicates performed using two donor-derived cell lines per group. (* *p* < 0.05; ** *p* < 0.01; Student’s *t* test.) (**B**) The relative quantification of Nrf2 protein levels was performed by cell-based ELISA in iPSC-derived neurons from control (CTRL), FXS patients, and UFM carriers. Nrf2 protein levels are normalized on GAPDH. Data are presented as the mean ± SEM of 7 independent experiment replicates using the same two donor-derived cell lines per group. (** *p* < 0.01 vs. CTRL; Student’s *t* test.)

To further characterize the functional context associated with altered SOD2 expression, intracellular reactive oxygen species (ROS) levels were measured using the DCFDA Cellular ROS Detection Assay Kit, which is a fluorometric assay (Figure 8A). Neurons derived from both UFM carriers and FXS patients (at 30 days of differentiation) exhibited significantly reduced ROS levels compared with control neurons. Specifically, UFM-derived neurons displayed a moderate but statistically significant decrease in ROS levels, whereas a more pronounced reduction was observed in FXS-derived neurons (Figure 8A).

Given the involvement of redox signaling in mitochondrial function, the transcription factor Nrf2 was subsequently assessed by ELISA (Figure 8B). Nrf2 protein levels were significantly reduced in neurons derived from both FXS patients and UFM carriers compared with control neurons. Quantitative analysis revealed a marked decrease in Nrf2 protein levels in FXS-derived neurons, as well as in both UFM-derived neuronal lines, with all patient groups displaying significantly lower Nrf2/GAPDH ratios relative to controls. Notably, reduced Nrf2 protein levels were observed in parallel with decreased intracellular ROS levels in patient-derived neurons. These findings indicate a correlation between altered Nrf2 protein levels and redox status in FXS and UFM neurons; however, given that Nrf2 was assessed only at the protein level, these data do not allow for conclusions regarding Nrf2 transcriptional activity or pathway activation.

## 4. Discussion

The present study demonstrates that mitochondrial dysfunction is a prominent feature of neurons derived from iPSCs carrying distinct *FMR1* mutation states. By extending our previous observations in patient-derived fibroblasts [7,8], we show that both FXS and UFM neurons exhibit alterations in mitochondrial architecture, bioenergetics, and redox homeostasis, although with distinct genotype-specific signatures that evolve during neuronal differentiation.

One of the most striking findings of this study is the presence of pronounced mitochondrial morphological abnormalities, particularly in UFM-derived neurons. Confocal and ultrastructural analyses revealed frequent donut-shaped mitochondria, altered mitochondrial elongation, and abnormalities in crista organization. Donut-shaped mitochondria are increasingly recognized as markers of mitochondrial stress and altered fusion–fission dynamics, frequently associated with disturbances in calcium homeostasis, oxidative stress, and bioenergetic dysfunction [25,26,27,28,29,30,31]. The observation that similar mitochondrial alterations were previously identified in fibroblasts from PM, UFM, and FXS individuals [7,8] suggests that mitochondrial structural remodeling may represent a common consequence of *FMR1* dysfunction across multiple cell types. The quantitative analysis of mitochondrial fraction revealed a genotype-dependent pattern. At 30 days, the mitochondrial fraction differed significantly among groups, with FXS neurons showing a marked reduction compared with controls, whereas UFM neurons displayed a significant increase. These opposite changes suggest distinct mitochondrial remodeling processes during early neuronal differentiation. In FXS neurons, the reduced mitochondrial fraction may reflect impaired mitochondrial maintenance, distribution, or turnover, potentially limiting energetic support during neuronal development. Conversely, the increased mitochondrial fraction observed in UFM neurons may indicate the compensatory expansion of the mitochondrial network or persistence of structurally altered mitochondria rather than an increase in fully functional organelles. The apparent discrepancy between the increased MitoTracker-positive mitochondrial fraction observed in UFM neurons and the reduced number of mitochondria detected by TEM likely reflects the different nature of the two measurements: confocal analysis estimates the area occupied by the mitochondrial signal, whereas TEM quantifies discrete organelle profiles. Thus, enlarged, elongated, clustered, or morphologically altered mitochondria may increase the mitochondrial area fraction despite the reduced number of individual mitochondrial profiles. At 60 days, differences in the mitochondrial fraction remained significant but were less pronounced, suggesting partial convergence during neuronal maturation. However, this apparent normalization does not necessarily indicate the restoration of mitochondrial homeostasis, as neuronal mitochondrial function depends not only on abundance but also on morphology, quality control, intracellular distribution, and local metabolic capacity. The observation that the levels of key mitochondrial enzymes (i.e., CS, OXPHOS), or transporters (i.e., ANT), are not in line with the mitochondrial fraction might suggest that a high number of mitochondria does not necessarily align with good function in FXS or UFM neurons.

Interestingly, mitochondrial defects were not uniformly distributed throughout neuronal compartments. UFM neurons exhibited a marked depletion of mitochondria in apical and perinuclear regions, whereas FXS neurons displayed milder alterations. Since these compartments are critically involved in synaptic input integration and mitochondrion–nucleus communication, respectively, their selective vulnerability may have important functional implications. Proper mitochondrial trafficking and compartmentalization are essential for neuronal development and synaptic plasticity, and their disruption has been implicated in several neurodevelopmental and neurodegenerative disorders [30,31,32]. The pronounced compartment-specific defects observed in UFM neurons therefore suggest that residual FMRP expression in this genotype is insufficient to fully preserve mitochondrial distribution and intracellular energetic homeostasis. The analysis of mitochondrial ultrastructure further revealed significant alterations. Crista architecture is a major determinant of respiratory efficiency because respiratory chain complexes and ATP synthase are highly enriched within crista membranes [33,34,35,36,37]. The increased crista density observed in the central regions of both FXS and UFM neurons may represent a compensatory response to impaired mitochondrial function, whereas the reduced number of mitochondria detected in apical compartments might reflect local bioenergetic insufficiency. Such structural adaptations are consistent with the hypothesis that mitochondrial remodeling serves as an attempt to maintain ATP production under conditions of chronic cellular stress.

Consistent with the ultrastructural findings, biochemical analyses revealed differentiation-dependent alterations in mitochondrial OXPHOS complexes. FXS neurons displayed a modest increase in OXPHOS protein abundance, whereas UFM neurons showed a marked reduction at early stages of differentiation, followed by normalization during neuronal maturation. Despite these differences in protein expression, both genotypes exhibited impaired mitochondrial respiration. Seahorse analyses demonstrated reductions in basal and maximal oxygen consumption rates together with a blunted response to FCCP, indicating reduced respiratory reserve capacity. These findings suggest that the observed increase in OXPHOS protein levels in FXS neurons does not translate into improved respiratory performance. This apparent discrepancy may reflect the presence of non-functional OXPHOS protein complexes, as previously suggested for the ATP synthase subunit c [35,38], or may represent a compensatory response to underlying mitochondrial dysfunction. However, these possibilities remain speculative, and further studies are required to determine whether reduced FMRP levels directly contribute to these alterations.

The discrepancy between OXPHOS protein abundance and respiratory activity is particularly evident in FXS neurons, where elevated levels of respiratory chain components coexist with impaired mitochondrial respiration. Similar mismatches between respiratory protein content and mitochondrial performance have been reported in several neurodegenerative conditions and are often interpreted as evidence of compensatory mitochondrial biogenesis or the defective assembly of respiratory super complexes [39,40,41,42,43]. Our findings therefore suggest that neuronal mitochondria carrying *FMR1* mutations are characterized by qualitative rather than purely quantitative defects in oxidative phosphorylation. An important mechanistic insight emerging from this study concerns the expression of proteins involved in the regulation of the mitochondrial PTP. The PTP is a high-conductance channel of the inner mitochondrial membrane whose prolonged opening promotes mitochondrial depolarization, matrix swelling, respiratory failure, and cell death [11,12]. Increasing evidence indicates that pathological PTP activation contributes to neuronal dysfunction and neurodegeneration [44,45,46,47]. In the present study, UFM neurons exhibited persistently elevated cyclophilin D (CyPD) levels together with reduced ANT, ATP synthase subunit and IF1 expression during early differentiation. Since CyPD sensitizes mitochondria to PTP opening [16], whereas ANT, ATP synthase and IF1 are important modulators of mitochondrial bioenergetics and permeability transition [11,41,42,43], this expression pattern might suggest the increased susceptibility of UFM neurons to permeability transition. These findings are particularly relevant considering our previous demonstration that fibroblasts carrying FXS and UFMs exhibit increased calcium-dependent PTP opening and enhanced mitochondrial-mediated apoptosis [8]. The persistence of elevated CyPD expression in differentiated UFM neurons suggests that altered PTP regulation might represent a conserved pathogenic mechanism linking mitochondrial dysfunction to neuronal vulnerability in fragile X-related disorders.

Another notable observation is the absence of increased mitochondrial swelling in patient-derived neurons despite molecular signatures consistent with enhanced PTP sensitivity. One possibility that might explain this apparent discrepancy resides in the unique metabolic demand of neurons. Due to their elevated oxidative metabolism and limited glycolytic reserve, neurons are particularly vulnerable to mitochondrial damage and may rapidly undergo apoptosis following permeability transition, thereby reducing the likelihood of detecting swollen mitochondria in surviving cells by TEM in situ [45,46,47]. Thus, the lack of observable swelling does not necessarily exclude enhanced PTP activity but might indicate a more rapid progression toward cell death, as shown in fibroblasts derived from patients [8]. Future studies will help to clarify these aspects that need further efforts to establish reliable methods to measure PTP opening and apoptosis in adherent iPSC-derived neurons.

The redox analyses performed in this study revealed an unexpected decrease in intracellular ROS levels in both FXS and UFM neurons, accompanied by increased SOD2 expression and reduced Nrf2 protein levels. Although DCFDA does not discriminate among individual ROS or selectively assess mitochondrial ROS, in the present study it was used to evaluate the overall intracellular oxidative status. Accordingly, these findings should be interpreted in terms of changes in cellular redox homeostasis rather than as a direct measurement of mitochondrial oxidative stress. Future studies employing more specific approaches, including mitochondrion-targeted probes such as MitoSOX, will be important to specifically investigate mitochondrial ROS production in FXS and UFM neurons. These observations partially recapitulate our previous findings in fibroblasts [7], where elevated SOD2 levels were associated with reduced oxidative burden. SOD2 represents the primary mitochondrial antioxidant enzyme responsible for the detoxification of superoxide radicals generated by oxidative phosphorylation [48,49,50]. The increased SOD2 expression observed in FXS neurons is consistent with an adaptive antioxidant response, although the present data do not establish whether this change contributes to the lower intracellular ROS levels. The concomitant reduction in Nrf2 is particularly intriguing because Nrf2 is generally activated under conditions of oxidative stress [51,52,53]; however, its relationship with the observed ROS levels remains unclear, as Nrf2 expression and activity can be influenced by multiple regulatory mechanisms.

The redox analyses revealed a decrease in intracellular ROS and Nrf2 levels in both FXS and UFM neurons. However, a greater increase in the SOD2 protein was observed exclusively in FXS cells, while UFM neurons showed only an upward trend. Thus, the observed changes in SOD2 do not fully parallel the ROS phenotype, particularly in UFM neurons, suggesting that additional mechanisms contribute to the altered redox state. Furthermore, it is well known that the DCF probe serves as a broad indicator of global redox status rather than mitochondrial-specific species: future studies using ratiometric, targeted probes will be essential to map specific radical dynamics within these models.

Alternatively, altered FMRP-dependent regulation may contribute to changes in Nrf2 expression; however, this possibility remains speculative and requires further investigation. Further studies will be required to clarify the mechanistic relationship between FMRP deficiency, antioxidant responses, and redox signaling.

Importantly, our findings also highlight significant differences between fibroblasts and neurons carrying *FMR1* mutations. Whereas fibroblasts maintained relatively preserved respiratory function despite mitochondrial abnormalities [8], neurons exhibited clear defects in oxidative phosphorylation and glycolytic capacity. This observation underscores the importance of studying disease-relevant neuronal models, as the exceptionally high energetic demands of neurons may unmask mitochondrial defects that remain compensated in peripheral cell types. Such neuron-specific vulnerability is consistent with the central role of mitochondrial metabolism in synaptic transmission, neuronal maturation, and cognitive function.

However, our study has some limitations, including the relatively small number of patient-derived neuronal lines and the inherent variability associated with the iPSC differentiation protocol. Moreover, although our findings support mitochondrial dysfunction as a conserved phenotype across cell types, further studies using additional patient-derived lines and complementary functional approaches will be required to establish the generalizability and underlying mechanisms of these alterations. An additional limitation concerns the assessment of oxidative status using the DCF assay, which provides a broad measure of intracellular oxidation but does not specifically quantify mitochondrial ROS or identify their source. This assay was selected as a direct and readily available approach, considering the technical challenges associated with obtaining sufficient numbers of iPSC-derived neurons for more specialized analyses. Future studies using mitochondrion-targeted ROS probes, such as MitoSOX, or complementary approaches to assess oxidative damage will be required to further characterize these processes.

## 5. Conclusions

Mitochondrial dysfunction emerges as a prominent feature of both FXS and UFM neurons and reveals that alterations in mitochondrial structure, respiratory capacity, PTP regulatory pathways, and redox homeostasis are maintained in disease-relevant neuronal cells. Despite the shared impairment of mitochondrial function, FXS and UFM neurons show genotype-specific patterns at early stages of differentiation, suggesting that different mechanisms might alter mitochondrial bioenergetics in patients. The phenotype observed in UFM neurons suggests that the partial preservation of FMRP expression does not necessarily confer protection against mitochondrial dysfunction. Together, these findings support the hypothesis that mitochondrial impairment represents a convergent pathogenic mechanism across fragile X-related disorders and suggest that the modulation of mitochondrial bioenergetics and PTP opening may represent promising therapeutic targets for future intervention strategies.

## Data Availability

The data supporting the findings of this study are available within the article and its Supplementary Materials.

## Supplementary Materials

The following supporting information can be downloaded at https://www.mdpi.com/article/10.3390/antiox15101295/s1, Figure S1: Complete characterization of neurons differentiated from iPS cells. Figure S2. Representative Laurdan and MitoTracker staining in differentiated neurons (Ctrl and UFM1).
