# Supplementary material for: Mitochondrial Remodeling and Impaired Bioenergetics Define Neuronal Vulnerability in Fragile X-Related Disorders
Source: Antioxidants (Basel). 2026 Oct 5;15(10):1295. doi: 10.3390/antiox15101295 (PMC13700763; doi:10.3390/antiox15101295)
Supplement: Supplementary file 1 [file antioxidants-15-01295-s001.zip › antioxidants-4514636-supplementary.pdf]

Supplementary data

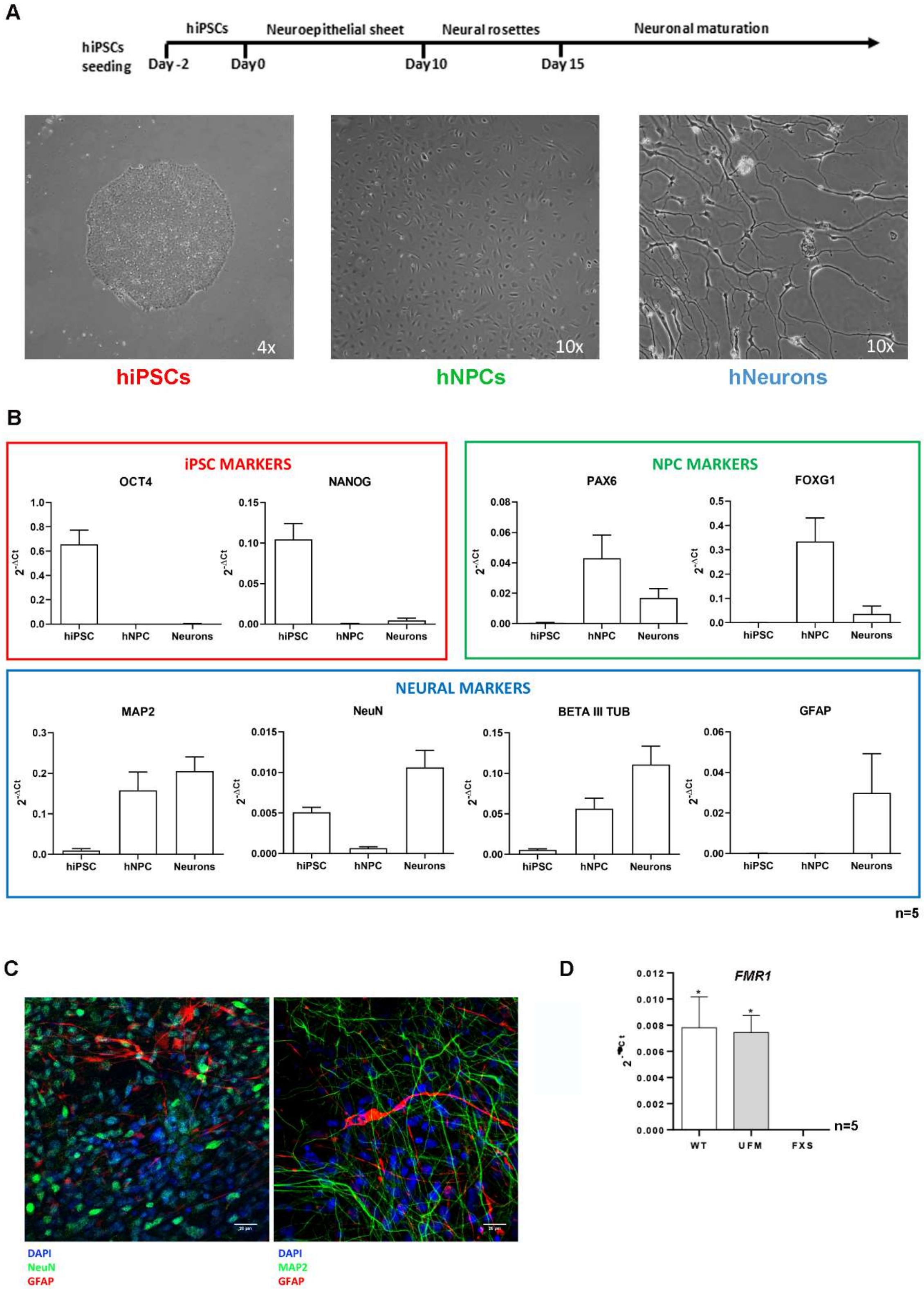

Figure S1. Complete characterization of neurons differentiated from iPS cells. (A) Images at optical microscopy of iPSCs at different stages of neuronal differentiation, as indicated by the timeline above.

(B) Quantification through qPCR of transcriptional markers at staminal stage (red), neuronal precursor (blue) and neuronal differentiation at 30 days of differentiation (green). (C) Representative images at confocal microscopy of differentiated neurons at 30 days stained for specific markers. NeuN (on the left), a nuclear neuronal marker, and MAP2 (on the right), a cytoplasmatic neuronal marker, are both stained in green. Nuclei are counterstained with DAPI (blue), while GFAP (a glial marker) is stained in red. Scale bar, 10 µm. (D) Quantification through qPCR of *FMR1* transcript after differentiation of iPS into neurons (30 days). Note the absence of *FMR1*-mRNA in FXS neurons compared to UFM. *p<0.05 vs. FXS (One-way ANOVA followed by Holm–Šídák post hoc test) n = 5 independent experiments.

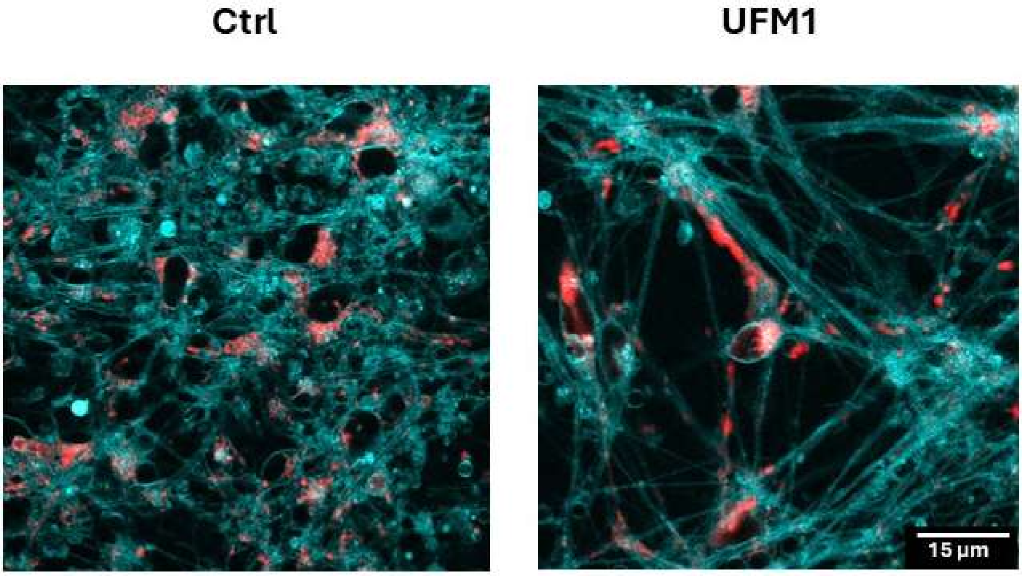

Figure S2. Representative Laurdan and MitoTracker staining in differentiated neurons (Ctrl and UFM1). Laurdan (cyan) was used to visualize cellular morphology and define cell boundaries, whereas mitochondria were labelled with MitoTracker™ Red (red). Scale bar, 15 µm.
